# Codon optimality modulates cellular stress and innate immune responses triggered by exogenous RNAs

**DOI:** 10.1101/2024.11.26.625518

**Published:** 2024-11-26

**Authors:** Chotiwat Seephetdee, Daniel L. Kiss

**Affiliations:** 1 Center for RNA Therapeutics, 6670 Bertner Ave, Houston, TX 77030 USA; 2 Department of Cardiovascular Sciences, 6670 Bertner Ave, Houston, TX 77030 USA; 3 Houston Methodist Academic Institute, 6670 Bertner Ave, Houston, TX 77030 USA; 4 Weil Cornell Medical College, 6670 Bertner Ave, Houston, TX 77030 USA; 5 Houston Methodist Cancer Center, 6670 Bertner Ave, Houston, TX 77030 USA; 6 Houston Methodist Research Institute, 6670 Bertner Ave, Houston, TX 77030 USA

## Abstract

The COVID-19 mRNA vaccines demonstrated the power of mRNA medicines. Despite advancements in sequence design, evidence regarding the preferential use of synonymous codons on cellular stress and innate immune responses is lacking. To this end, we developed a proprietary codon optimality matrix to re-engineer the coding sequences of three luciferase reporters. We demonstrate that optimal mRNAs elicited dramatic increases in luciferase activities compared to non-optimal sequences. Notably, transfecting an optimal RNA affects the translation of other RNAs in the cell including control transcripts in dual luciferase assays. This held true in multiple cell lines and for an unrelated reporter. Further, non-optimal mRNAs preferentially activated innate immune pathways and the phosphorylation of the translation initiation factor eIF2α, a central event of the integrated stress response. Using nucleoside-modified or circular RNAs partially or fully abrogated these responses. Finally, we show that circularizing RNAs enhances both RNA lifespan and durability of protein expression. Our results show that RNA sequence, composition, and structure all govern RNA translatability. However, we also show that RNA sequences with poor codon optimality are immunogenic and induce cellular stress. Hence, RNA sequence engineering, chemical, and topological modifications must all be combined to elicit favorable therapeutic outcomes.

## Introduction

As demonstrated by the efficacy of messenger RNA (mRNA) vaccines against severe coronavirus disease 2019 (COVID-19), mRNA has emerged as a crucial therapeutic modality in medicine (1,2). With applications in cancer immunotherapy, regenerative medicine, and the treatment of genetic disorders, mRNA therapies are expanding to indications beyond infectious diseases (3–5). Several innovative approaches have been shown to advance mRNA technology by reducing immunogenicity and/or by enhancing mRNA stability and translation efficiency. Substitution of uridine (U) with *N*^1^-methylpseudouridine (m1Ψ) minimizes inherent mRNA immunogenicity by dampening recognition by Toll-like receptors (TLRs) of the innate immune system while also markedly increasing protein expression (6). Coding sequence (CDS) and untranslated region (UTR) optimization has also been shown to enhance protein production (7-9). The development of circular RNA (circRNA) and self-amplifying mRNA, which prolong RNA lifespan and translation durability due to exonuclease resistance and self-replication ability respectively, are major advances that extend mRNA pharmacokinetics (10-14). Further, RNA circularization also decreases the immunogenicity of unmodified mRNAs, in part due to TLR evasion (11,15). Recently, through chemo-topological engineering efforts, mRNA lifetime and translation capacity have also been augmented by multimerizing the poly(A) tail and by chemical modifications of the 5’ end’s 7-methylguanosine cap structure (16,17). These continuous improvements evince the potential for building better mRNA therapeutics.

Despite the advancements observed in mRNA as a platform technology, the impact of open reading frame (ORF) sequences on regulating gene expression remains under-reported, especially in the context of protein-coding RNA therapeutics, in the published literature. For instance, synonymous codon usage, is one way to modulate gene expression by regulating translation, mRNA stability, and mRNA localization (18,19). The degeneracy in the genetic code means that multiple distinct triplet codons code for most amino acids. During translation elongation, the ribosome recognizes synonymous codons differently, leading to local changes in translation rates within the CDS of an mRNA. The stochastic recognition of the codon at the ribosome A-site by charged tRNAs, which contributes to ribosome elongation dynamics, is also influenced by, among other things, the abundance of the cognate tRNA and wobble (non-Watson-Crick) base pairing. New evidence has also implicated selected arginine tRNAs for co-translational P-site tRNA-mediated mRNA decay (PTMD) pathway mediated by CNOT3 (20). A sustained series of non-optimal codons is thought to locally slow or pause translation elongation, sometimes leading to ribosome collisions and subsequent mRNA degradation (19,21). A growing body of work has highlighted the implication of ribosome collisions on integrated stress response and innate immune activation (22,23). Therefore, it is reasonable to postulate that codon optimality of exogenous mRNAs is relevant to these cellular events as well.

Codon optimization has become a common practice in heterologous gene expression (24). As a result, several indices have been employed to optimize the codon usage including the codon adaptation index (CAI) (25). The CAI provides an approximate indication of heterologous gene expression level by scoring the codon usage frequency of a given gene compared to that of a reference organism (25). Although based on genomic sequences, the CAI score can also be used to approximate codon optimality. For example, the COVID-19 mRNA vaccines represent an obvious example of the superiority of optimal codon usage in an RNA therapeutic. The CAI score of the native SARS-CoV-2 S protein is 0.67 while CAI scores of mRNA-1273 and BNT162b2 vaccines are 0.98 and 0.95, respectively (26-28). Since mammals have a bias for G/C at wobble positions, codon optimization for human therapeutics tends to increase GC content, thereby reducing U content (29). As a result, high GC content and strong RNA secondary structure can influence both translation rate and efficiency (30). Furthermore, unmodified mRNAs with reduced U content could minimize innate immune activation, thereby increasing protein expression (7,8).

In this work, we hypothesized that the codon optimality of exogenous mRNAs can influence or modulate cellular stress and/or innate immune responses. We re-engineered the coding sequence of three luciferase genes, including Nanoluciferase (NLuc), *Renilla* luciferase (RLuc), and firefly luciferase (FLuc) using a proprietary codon optimality matrix. In all cases, the engineered, optimal reporter mRNAs achieved superior luciferase activity compared to the non-optimal sequences. Moreover, we observed that the optimality of the exogenous test mRNAs also had an effect on the expression of the transfection control mRNAs. In particular, non-optimal mRNAs suppressed the expression of the independent control reporter compared to optimal mRNAs. We further interrogated the effects of mRNA codon usage on possible mechanisms contributing this observation. Specifically, we show that non-optimal mRNAs led to a substantial activation of the eukaryotic translation initiation factor α (eIF2α) phosphorylation, which is a hallmark of integrated stress response that globally suppresses translation initiation. Intriguingly, we demonstrate that substitution of non-optimal codons with synonymous, optimal codons significantly reduced eIF2α phosphorylation levels. Further, we demonstrate that codon optimality also contributes to the immunogenicity of mRNA. Our data reveal that non-optimal mRNAs strongly induced the expression of innate immunity genes which is markedly reduced both by using m1Ψ and by substituting an mRNA with more optimal codons. Lastly, by utilizing destabilized luciferase reporters, we demonstrate that circular RNAs with optimal coding sequences also have an extended lifespan. Taken together, our findings indicate that engineering coding sequences to increase codon optimality provides an additional therapeutic mRNA design approach to modulate /minimize cellular stress and innate immune responses.

## Materials and methods

All materials, reagents, their suppliers, and catalog numbers are included in Table S1: Key Resources.

### Plasmid construction

The template plasmids used for in vitro transcription of linear and circular mRNAs encoding non-optimal luciferase reporters were constructed using e-Zyvec’s (Polyplus-Sartorius) whole plasmid synthesis service. The linear mRNA template consists of a T7 promoter, 5’ UTR, NcoI and XhoI cloning sites for inserting the ORFs, 3’ UTR, 90-nt poly(A) tail, followed by BspQI restriction site. The circRNA template consists of a T7 promoter, homology regions, spacers and permuted *Anabaena* pre-tRNA group I intron-exon sequence on 5’ and 3’ ends, CVB3 IRES and the coding region and is adapted from the literature (10,11). The optimal reporter gene fragments were synthesized by Twist Bioscience and subcloned into linear mRNA and circRNA vectors by ligation and HiFi DNA assembly, respectively. To generate optimal linear mRNA templates, the linear mRNA backbone and gene fragments were digested by NcoI and XhoI followed by ligation. The circRNA backbone was PCR amplified using primers CS31 and CS32 (see Table S2 for all primer sequences). Gene fragments were PCR amplified using primers CS33 and CS34. The amplified products were gel purified and assembled by NEBuilder HiFi DNA Assembly master mix. All linear mRNA and circRNA vectors were transformed into NEB stable competent *E. coli* and NEB 5-alpha competent *E. coli*, respectively. The plasmids were miniprepped with Monarch Plasmid Miniprep Kit and sequence-verified with whole-plasmid sequencing. For linear mRNA templates, the length of poly(A) tail was verified by Sanger sequencing. All novel plasmids will be submitted to Addgene (Accession number pending) upon peer-reviewed publication of this work.

### In Silico RNA Sequence assessment

The minimum free energy (MFE) was calculated from the predicted RNA secondary structure using RNAFold v2.7.0 at the default settings. The codon adaptation index (CAI) was calculated using CAI calculator on https://www.biologicscorp.com.

### mRNA synthesis and purification

Unmodified or modified linear mRNAs were synthesized by *in vitro* transcription from a linearized plasmid DNA template and co-transcriptionally capped using a HiScribe^®^ T7 mRNA Kit with CleanCap Reagent AG Kit according to the manufacturer’s instructions. For modified linear mRNAs, UTP was replaced by *N*^1^-methylpseudo-UTP. circRNA precursors were synthesized by *in vitro* transcription from a linearized plasmid DNA template using a HiScribe T7 High Yield RNA Synthesis Kit according to the manufacturer’s instructions. The *in vitro* transcription reactions were treated with DNase I to remove residual DNA template and RNAs were purified by Monarch Spin RNA Cleanup Kit . To circularize the RNA, GTP was added to a final concentration of 2 mM along with the circularization buffer containing 50 mM Tris-HCl, 10 mM MgCl2, 1 mM DTT, pH 7.5. RNA was then heated at 55 °C for 8 min. The circRNA products were further purified using a Monarch Spin RNA Cleanup Kit. To remove leftover linear RNA species, the purified circRNA products were treated with 0.5 U of RNase R per 1 μg of circularized RNA at 37°C for 1 h. The quality and quantity of RNA were evaluated by Denovix DS-11 FX+ Spectrophotometer and FlashGel RNA Cassettes.

### Cell culture and transfection

HEK293 and NIH/3T3 cells were cultured in Dulbecco’s modified Eagle’s medium (DMEM) plus 10% FBS; U2OS cells in McCoy’s 5A plus 10% FBS; BJ cells in Eagle’s Minimum Essential Medium (EMEM) plus 10% FBS. All cells were maintained in a 37°C incubator with 5% CO2 and passaged approximately every 3 days. mRNAs were transfected with Lipofectamine MessengerMAX Transfection Reagent at equi-molar concentrations unless stated otherwise along with an internal control mRNA per the manufacturer’s protocols. The transfection reagent was removed, and fresh medium was added to the cells 6 h after transfection.

### Luciferase assays

Cells were lysed with Passive Lysis Buffer at the indicated time points after transfection. For Firefly/*Renilla* assays, luciferase activity was measured using Dual-Glo Luciferase Assay System. For Firefly/NanoLuc assays, luciferase activity was measured using Nano-Glo Dual Luciferase Reporter Assay System. Luminescence was measured on a white 96-well flat-bottom microplate using a SpectraMax iD5 Microplate Reader (Molecular Devices).

For protein half-life determination, 24 h after transfection, cells were either lysed with the passive lysis buffer and frozen at −20°C (for T0 control) or treated with 100 μg/mL CHX (Sigma, C1988) for the indicated amounts of time before lysis/freezing. Luciferase assays were performed on thawed samples using the appropriate dual-reporter assay system mentioned above.

For determining proteasome-mediated protein degradation, cells were transfected with degron-tagged reporter mRNAs for 16 h. Subsequently, cells were transfected with the optimality reporter mRNAs and treated with DMSO (for control) or 10 μg/mL MG-132 (Sigma, 474787). After 6 h, cells were lysed with passive lysis buffer and luciferase assays were performed using the corresponding dual-reporter assay system.

### Live fluorescence microscopy imaging

An Incucyte S3 Live-Cell Analysis System (Sartorius) was used to measure and quantify the levels of EGFP control mRNA expression. Four independently transfected wells were used to measure each time point and condition. For each well, five images were acquired at time points indicated after transfection for each signal determination. The signals for each image were quantified using the Incucyte S3 software (Incucyte 2022B Rev2), averaged for each well, and are shown as the mean fluorescence intensity.

### Western blot analysis

Sixteen hours after the transfection, media was removed, and cells were washed with phosphate-buffered saline (PBS). Lysates were collected by scraping using NP-40 lysis buffer (50 mM Tris, pH 8.0, 150 mM NaCl, 1% NP-40) with protease and phosphatase inhibitors. Proteins were collected from the supernatant after centrifugation at 16,000 x g for 20 min. Protein concentrations were determined using the Pierce BCA protein assay kit using a bovine serum albumin standard curve. Protein samples were then prepared in Laemmli sample buffer containing 50 mM DTT and heated at 95°C for 5 min. Samples were run on a 12% Mini-PROTEAN TGX Precast Protein Gel with Tris/Glycine/SDS buffer. The protein was transferred to a 0.2 μM PVDF membrane using Trans-blot Turbo Transfer System according to the manufacturer’s instructions. The membrane was blocked with TBST with 5% BSA and sustained rocking at room temperature for 1 h. The following antibodies were used: phosphorylated eIF2α (Ser51) (1:1000 dilution), eIF2α (1:1000 dilution), α-tubulin (1:20000 dilution), HRP-conjugated goat anti-rabbit IgG (1:5000 dilution), HRP-conjugated horse anti-mouse IgG (1:5000 dilution). Primary antibodies were diluted in TBST with 5% BSA, and blots were incubated rocking overnight at 4°C. Membranes were washed three times with TBST and incubated with secondary antibodies with rocking at room temperature for 1 h. Membranes were washed three times with TBST, and protein signal was detected with the Clarity Western ECL Substrate and imaged on the ChemiDoc MP Imaging System (Bio-Rad).

### RT-qPCR

Total RNA was extracted from cells by adding TRI Reagent to the cells and RNA was extracted by phase separation and isopropanol precipitation per the manufacturer’s instructions. One μg of total cellular RNA was treated with DNase I and reverse transcribed with random hexamer primers and ProtoScript II Reverse Transcriptase. RT-qPCR was performed with SSOAdvanced Universal SYBR Green Supermix on a CFX96 Touch Real-Time PCR Detection System. Each sample was measured in technical triplicates and three biological replicates were performed per experiment. Relative gene expression was calculated by the 2^−ΔΔCt^ method using β-actin as an internal control.

## Results

### The optimality of test mRNAs affects protein output from both test and control mRNAs

**Figure 1.**
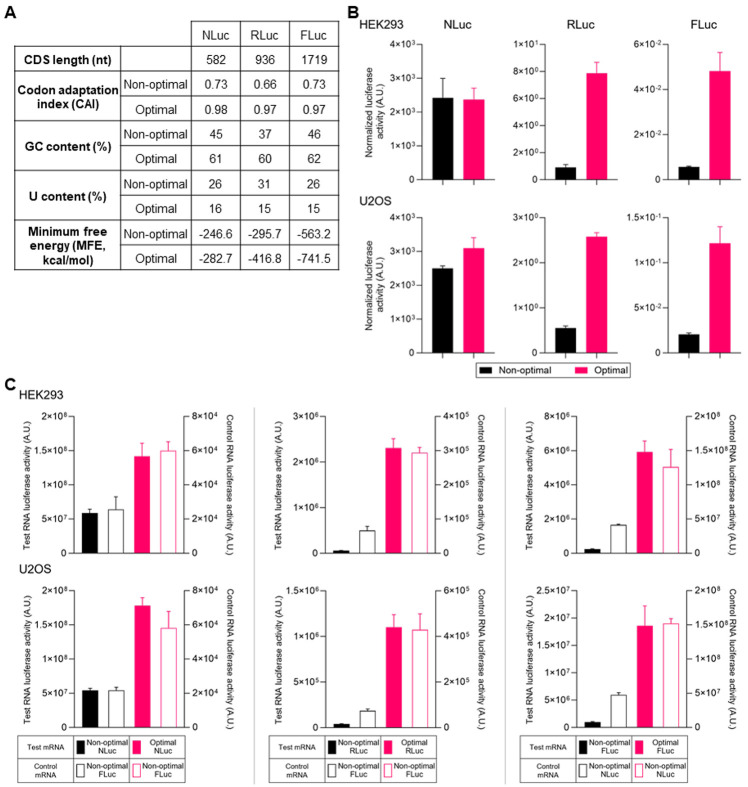
The codon optimality of test mRNAs affects expression of both test and control mRNAs. **(A)** Characteristics of non-optimal and optimal reporter coding sequences used in this study. Codon adaptation index (CAI) scores and minimum free energy (MFE) were calculated as described in methods. **(B)** Normalized luciferase activity from HEK293 and U2OS cells lysed 24 h after co-transfection with mRNAs encoding either optimal or non-optimal forms of Nanoluciferase (NLuc), *Renilla* luciferase (RLuc), or firefly luciferase (FLuc) reporters. Protein expression was measured by dual luciferase assays and the luminescence of optimal or non-optimal test reporters were normalized to control reporter luminescence. **(C)** Codon optimality of test mRNAs show differences in protein levels of both test and transfection control mRNAs. Dual-luciferase assays from HEK293 and U2OS cells lysed 24 h after transfection with mRNAs encoding either optimal or non-optimal forms of NLuc, RLuc, or FLuc test reporters (left axis, solid bars) along with non-optimal luciferase transfection control RNA (right axis, empty bars). Each experiment consists of a color-matched solid bar (read on the left Y-axis) and the empty bar (read on the right Y-axis) directly adjacent to it. Data are shown as the mean +Std Dev of four independent experiments.

To increase reporter protein expression from exogenous mRNAs, we developed and used a proprietary codon optimality matrix to re-engineer the coding sequences of NLuc, RLuc*,* and FLuc. Although there is no consensus regarding a unified codon optimality score, we used the human codon adaptation index (CAI) score as an approximate measure of optimality (25). Depending upon the reporter, the resulting codon selections for each luciferase reporter corresponded to a CAI score of 0.98, 0.97, and 0.97 for NLuc (+0.25), RLuc (+0.31), and FLuc (+0.24), respectively (Figure 1A). Overall, the optimal reporters have increased GC content, reduced U content, and a lower minimum free energy suggesting a more stable tertiary structure (Figure 1A) (30). We in vitro transcribed linear mRNAs encoding the individual reporters with standard ribo-nucleotides and co-transfected them into multiple cell lines for dual luciferase assays. To our surprise, while the optimal RLuc and FLuc reporters had significant increases in luciferase activity when we normalized the signals to the co-transfected control RNA, the optimal NLuc reporters showed no difference in luciferase activity in HEK293 cells and only had a marginal increase on luciferase activity in U2OS cells (Figure 1B). However, upon closer examination of the raw, non-normalized data, we noticed that all optimal reporters showed large increases in luciferase activity when compared to non-optimal mRNAs in all four (HEK293, U2OS, NIH/3T3, and BJ) cell types assayed (Figures 1C and S1A, left Y-axis). Intriguingly, we found that the co-transfected control reporter in non-optimal test reporter-transfected cells showed substantially lower levels of luciferase activity driven from the control mRNA compared to the same mRNA in cells transfected with optimal test reporters (Figure 1C, right Y-axis). In other words, the optimality of the test mRNA was exerting an effect on the control mRNA, which made normalizing the data troublesome. This held true regardless of which pair of luciferase transcripts were used. To rule out that this observation was caused by experimental artifacts built into dual luciferase assays, we validated this observation by co-transfecting the optimal luciferase reporter pairs with mRNA encoding an EGFP reporter protein (Figure S1B). In agreement with the dual luciferase assays, cells co-transfected with non-optimal luciferase mRNAs showed lower EGFP fluorescence signals measured by an Incucyte live cell imaging system than cells transfected with optimal luciferase reporters (Figure S1B). Together, these data show that the codon optimality of experimental mRNAs influences translation from the co-transfected control mRNAs.

### Unmodified linear mRNAs with poor codon optimality induces eIF2α phosphorylation and innate immunity gene activation

**Figure 2.**
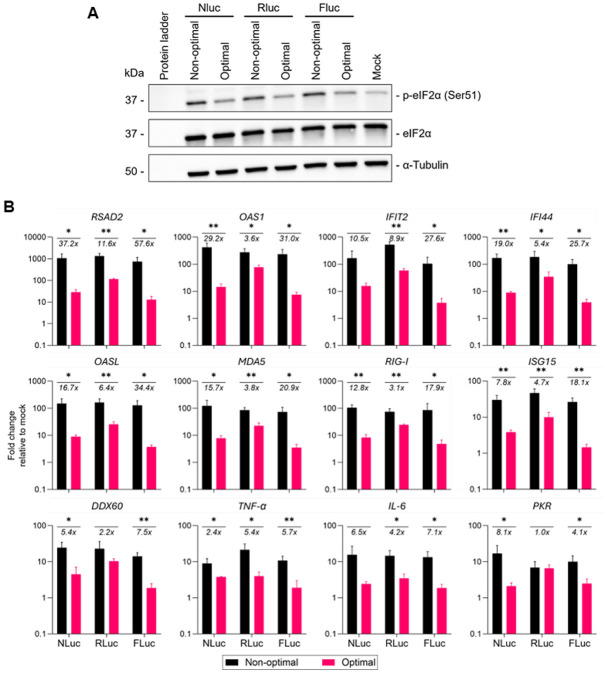
Codon optimality contributes to eIF2α phosphorylation and innate immune gene activation by unmodified linear mRNA. **(A)** HEK293 cells were harvested 16 h after transfection with mRNAs encoding either non-optimal or optimal forms of three luciferase reporters. Cell lysates were assayed by immunoblotting with antibodies targeting either phosphorylated eIF2α, unphosphorylated eIF2α, or α-tubulin (as a loading control). The levels of phosphorylated eIF2α in each condition were quantified by Image Lab and normalized to total eIF2α levels. **(B)** U2OS cells were transfected with reporter mRNAs encoding either non-optimal or optimal luciferase reporters for 16 h. Relative induction of innate immune genes were measured by qRT-PCR, with relative fold changes normalized to expression levels in mock transfected cells. Data are shown as the mean +Std Dev of three independent biological replicates. Student’s t-test was used to evaluate the significance of the results, and *p < 0.05, **p < 0.01.

In yeast, reporter mRNAs with non-optimal codons can lead to phosphorylation of the translation initiation factor eIF2α in a CDS length-dependent manner (31). Furthermore, a GCN2 homolog may preferentially inhibit translation elongation on mRNAs with long CDS enriched in non-optimal codons (31). The effect that codon usage has on eIF2α phosphorylation resembles the effect of ribosome stalling induced by translation elongation inhibitors (31). The resulting phosphorylated eIF2α leads to global reduction in translation and activates the integrated stress response pathways (22). We compared levels of eIF2α phosphorylation in HEK293 cells individually transfected with unmodified linear mRNAs encoding non-optimal or optimal reporters by Western blot analysis. Interestingly, cells transfected with non-optimal reporters exhibited a significant increase in eIF2α phosphorylation level over optimal mRNAs (Figure 2A). Notably, consistent with earlier observations, the intensity of eIF2α phosphorylation correlated with CDS length, with longer CDSs showing higher levels of eIF2α phosphorylation. The increased phosphorylated eIF2α levels are consistent with globally reduced (but not blocked) translation initiation and could explain the reduced control reporter expression observed in cells transfected with non-optimal mRNA.

Since exogenous unmodified linear mRNA is known to be immunogenic, we next asked whether differences in reporter protein expression was partially caused by reduced immunogenicity associated with optimal codon usage. We used RT-qPCR to assess the expression profile of a dozen innate immune genes, including interferon-stimulated genes (ISGs), antiviral response genes, inflammatory cytokines, and innate immune regulators in U2OS cells transfected with unmodified linear mRNAs encoding optimal or non-optimal reporter mRNAs. As expected, transfection of reporter mRNAs led to increased expression of innate immune response genes relative to mock transfection (Figure 2B). Notably, optimal mRNAs resulted in a significant suppression of innate immunity gene activation compared to the non-optimal reporters. These data show that poor codon optimality can contribute to the immunogenicity of exogenous mRNAs.

### Optimal codons increase reporter protein expression from linear and circular RNAs

**Figure 3.**
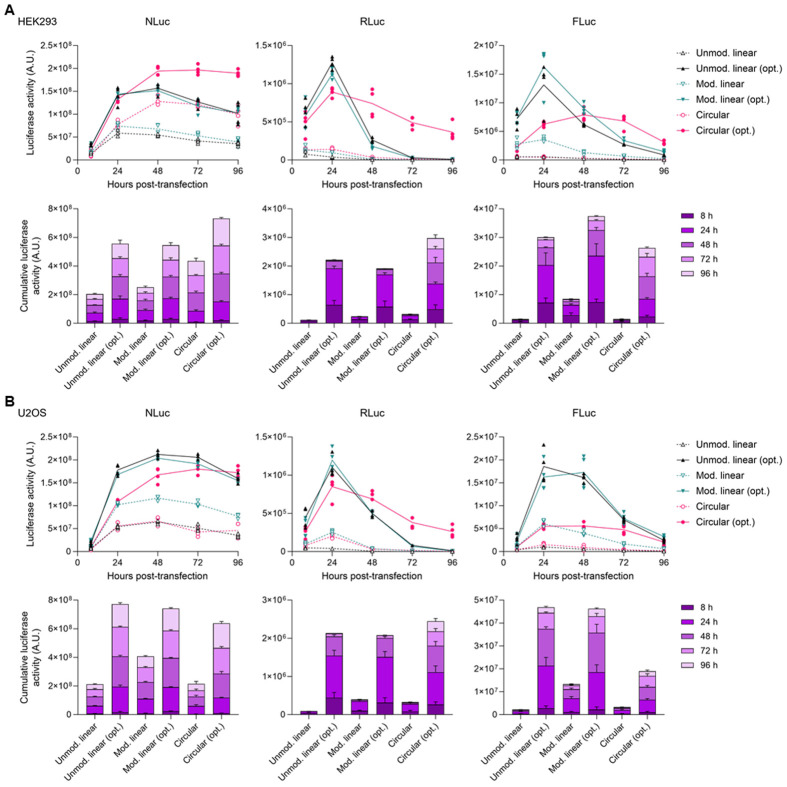
Optimal codons increase reporter protein expression from linear and circular RNAs. **(A)** HEK293 or **(B)** U2OS cells were transfected with unmodified linear, modified linear (100% m1Ψ substitution), or CVB3 IRES-driven circRNAs encoding either non-optimal or optimal forms of the indicated luciferase reporters. Cells were lysed and reporter protein expression was measured by the corresponding luciferase assay at 8, 24, 48, 72, and 96 h after transfection. The data are presented as a (top) time-course of luciferase activity over time and (bottom) as accumulated luciferase activity from 8 h to 96 h. Error bars represent Std Dev of four independent replicates.

mRNA engineering approaches, including chemical modification of nucleotides and RNA circularization, have also been shown to reduce immunogenicity while increasing the efficiency and durability of protein expression (6,10). We next evaluated the level and duration of protein expression from our optimality reporters delivered using three different RNA therapy platform technologies (unmodified mRNAs, modified mRNA, and circRNA). Consistent with the mRNA vaccines for COVID-19, modified linear mRNAs were made by complete substitution of U with m1Ψ (27,28). circRNAs containing a CVB3 IRES driving the translation of an optimal or non-optimal reporter CDS were generated by adapting a previously-published permuted intron-exon (PIE) strategy (10). Transfection into HEK293 and U2OS cells showed that optimal reporter mRNAs exhibited higher luciferase activities compared to non-optimal counterparts across both RNA types (Figure 3). We also observed different expression kinetics and durability of protein expression between linear and circular RNAs in which circRNAs predominantly showed lower peak level protein expression but maintained protein expression longer than linear mRNAs. The expression level and durability of expression also varied depending upon the reporter protein, but optimal sequences always outperformed non-optimal sequences for both parameters. Further, non-optimal circRNAs generally had similar expression kinetics and RNA half lives as non-optimal mRNAs.

Since incorporating modified nucleotides into mRNAs and circularization have been shown to reduce the immunogenicity of exogenous RNAs, we also tested to see how modified mRNA and circRNAs affected the expression of the transfection control mRNA, eIF2α phosphorylation, and innate immunity genes. The co-transfection of different optimality reporters encoding RLuc with an EGFP control mRNA in HEK293 and U2OS cells revealed that the reduction of EGFP signals observed in non-optimal unmodified linear mRNA transfection was abrogated when modified linear mRNA or circRNA were used (Figure S2A). Next, we analyzed the level of eIF2α phosphorylation in HEK293 cells individually transfected with unmodified linear, modified linear, or circRNAs encoding non-optimal or optimal RLuc reporters. Western blot analysis shows that the induction of eIF2α phosphorylation by non-optimal reporters was diminished when the identical CDS was delivered as either modified linear mRNA or circRNA (Figure S2B). The immunogenicity of different RNA platforms encoding optimality reporters was then compared by RT-qPCR analysis of transfected U2OS cells. The expression of innate immunity genes induced by non-optimal sequences was significantly blunted when delivered by nucleoside-modified mRNAs or circRNAs (Figure S2C). While m1Ψ substitutions abolished immunogenicity of linear mRNAs, unmodified circRNAs were still somewhat immunogenic, suggesting that cellular recognition of the natural nucleoside uridine and RNA ends is the major determinant of RNA immunogenicity. Another possibility is that trace amounts of circRNA precursors or processing intermediates (that are below the detection threshold of Flash Gels) drive the immune activation observed in our circRNA samples. Regardless, these data are consistent with each RNA therapy platform conferring unique immuno-stimulatory, or immune-avoidance, properties.

### RNA circularization increases the lifespan of optimal reporter transcripts in vivo

**Figure 4.**
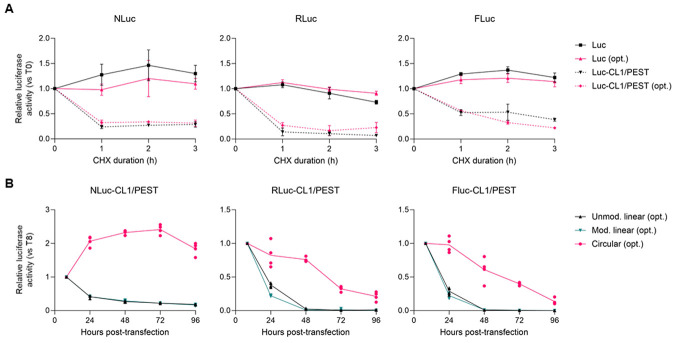
RNA circularization increases the lifespan of optimal reporter transcripts in HEK293 cells. Paired CL1/PEST degron motifs were fused to the indicated luciferase coding sequence to stimulate protein turnover (32). **(A)** HEK293 cells were co-transfected with linear mRNAs encoding a transfection control and the indicated reporters for 16 h. Luminescence signals were measured and normalized to the co-transfected control at the indicated time points after addition of cycloheximide (CHX, 100 μg/mL). The data shown for each reporter are the means ±Std Dev of four independent replicates and were compared to the time point when CHX was added (0 h). **(B)** HEK293 cells were co-transfected with a transfection control and either unmodified linear, modified linear, or circular mRNAs encoding optimal CL1/PEST degron-tagged luciferase reporters. Protein expression was measured by the corresponding luciferase assay at 8, 24, 48, 72, and 96 h after transfection. The data (shown as relative luciferase activity (RLU)) for each reporter are the means ±Std Dev of four independent replicates and were compared to the signal observed 8 h after transfection.

circRNAs have been touted as a promising next-generation RNA therapy platform since they are resistant to cellular exonucleases and confer longer in vivo lifespan than mRNA (10). Further, costly modified nucleotides and capping reagents are not required for circRNA production (11). To de-couple mRNA lifespan from the half-life of their encoded reporter proteins, we fused degron motifs to the C-terminus of each reporter protein to stimulate targeted degradation by proteasome (32,33). We estimated the half-life of our three luciferase reporter proteins in multiple cell lines by measuring luciferase activities of cells transfected with unmodified linear mRNA after adding the translation inhibitor cycloheximide (CHX) at different time points. Luminescence data showed that the reporters bearing degron motifs had ~8-fold lower signals than non-degron containing versions, and that the half-life of degron-bearing luciferase reporter proteins was ~1 hour or less (Figures 4A and S4A). Notably, at the protein level, both non-optimal and optimal reporters showed essentially identical half-lives, validating that the codon optimality of the RNA does not affect the stability of the encoded reporter protein. We then used an extended time-course to compare the luciferase activity of each destabilized reporter from unmodified linear, modified linear, and circular RNAs. As predicted, circRNAs encoding optimal degron-tagged reporters have longer half-lives than their linear mRNA counterparts regardless of whether they used modified bases (Figures 4B, S3, and S4B-C). We also observed the differences in the half-lives of RNAs encoding different reporters that correlated with the length of the CDS, with RNAs encoding shorter ORFs being longer-lived.

### Codon optimality of test mRNAs does not affect proteasome-mediated degradation

We next asked whether the codon optimality of the test RNAs influences global proteasome-mediated protein degradation which could conceivably cause the differences we observed in the expression of the transfection control reporter. We compared the degradation of degron-tagged reporters after treating two cell lines with the proteasome inhibitor MG-132 or DMSO as a control. Regardless of the reporter transfected, we did not observe significant differences in degradation rates of the control reporters after transfection with either non-optimal or optimal mRNAs (Figure S5).

Taken together, out data indicate that mRNAs enriched in non-optimal codons preferentially trigger cellular innate immune pathways and the phosphorylation of the translation initiation factor eIF2α, resulting in reduced global translation and at least a partial activation of cellular stress response. These responses were fully or partially abrogated when nucleoside-modified or circular RNAs were used. Finally, we show that circularizing an optimal CDS enhances both RNA lifespan and the durability of protein expression from exogenous RNAs.

## Discussion

Despite the tremendous success of RNA therapeutics in various areas of medicine, their inherent instability and temporally limited translation capacity in vivo limit their application to many indications. Many previous reports have delineated the consequences of codon optimality on the translational regulation and stability of mRNAs encoded by different mixes of synonymous codons (18,19,34-36). However, little has been published about the significance of codon optimality on cellular stress and innate immune responses with regards to exogenous RNAs. Elucidating this link is particularly timely since the use cases for RNA medicines will continue to expand, and such knowledge can drive the design of future therapies.

Here, we report that codon optimality is one determinant of mRNA immunogenicity and that mRNAs with sub-optimal sequences can activate cellular stress responses in mammalian cells. Using three different reporters, our data demonstrate that optimal mRNAs result in superior expression parameters (peak luciferase activity and expression duration) for all three luciferase reporter proteins tested. This is in line with previous attempts to enhance protein expression from mRNAs by optimizing the codon optimality and/or global mRNA secondary structure (34,37,38). To our surprise, our data show that optimal test reporters markedly increased the baseline luciferase activity of the co-transfected control mRNA. In other words, we observed that the codon optimality of test mRNAs changed the protein output from the transfection control mRNAs. This is a key observation as the transfection control of a dual reporter assay must (and is assumed to) remain unchanged to accurately capture the changes observed in these reporter experiments.

Our data also suggest that the optimality of transfected RNAs may drive changes in global translation regulation. We show that non-optimal mRNAs induce eIF2α phosphorylation, a key mediator of the integrated stress response that globally suppresses translation initiation. In line with our observations in mammalian cells, experiments in yeast also show that consistent use of sub-optimal codons induces eIF2α phosphorylation in a CDS length-dependent manner (31). This observation raises the question of whether the slower translation elongation rate attributed to non-optimal codons could lead to ribosome collisions, which have been reported to trigger several cellular stress response pathways (22). Earlier observations showed that substituting U with Ψ or m1Ψ leads to increased amino acid substitution by the ribosome in both prokaryotic and mammalian translation systems (39). While that earlier study did not directly assess ribosome collisions, a provocative hypothesis is that, on average, such m1Ψ-caused amino acid substitutions would shorten ribosome residence times at individual sub-optimal codons. In particular, this would have a marked effect on local regions of mRNAs enriched in low-optimality codons by helping reduce ribosome pausing and/or eliminate ribosome collisions on modified mRNAs. Our data show that eIF2α phosphorylation was diminished by substituting U with m1Ψ and are consistent with that hypothesis. As with including modified bases, our results are consistent with previous reports showing that RNA circularization also tempers eIF2α phosphorylation (15,40).

Further, we showed that codon optimality also contributes to the immunogenicity of exogenous RNAs. Specifically, non-optimal mRNAs led to the induction of several ISGs, antiviral response genes, inflammatory cytokines, and innate immune regulators. Notably, substitutions of non-optimal codons by synonymous, optimal codons significantly mitigate innate immune response activation by unmodified linear mRNAs. ISG activation was also fully or partially abrogated by using m1Ψ and circularizing the RNAs containing identical coding sequences. This effect might be partly due to the lower uridine content in optimal mRNAs, which impedes recognition by TLRs (7,8,41-44) and a reduction in double-stranded RNA by-products from m1Ψ-containing *in vitro* transcription reactions (45). This observation also suggests that crosstalk between ribosome pausing (or stalling) caused by non-optimal codons and innate immunity gene activation is possible (23).

Finally, we sought to learn how these different types of RNA therapeutics affected the half-lives of the RNAs in vivo. As such, we fused degron motifs onto the reporter proteins to induce rapid, proteasome-mediated protein turnover. With the reporter protein’s half-life reduced to <1 hour, this enabled us to use the persistence of the reporter protein’s signal to estimate the half-life of protein coding RNA itself (32,33). circRNAs encoding optimal reporters show ORF-dependent increased RNA half-lives compared to their linear mRNA counterparts. Differences in expression kinetics between linear and circular RNAs were observed, which is possibly due to distinct translation initiation mechanisms (cap-dependent vs IRES) and the distinct topology of circRNAs. The fact that circRNAs are slightly immunogenic led us to speculate whether different IRES sequences could be exploited to tailor immune activation for certain therapeutic purposes.

Collectively, the codon optimality and the platform technology of exogenous RNAs both play important roles in harnessing (or evading) cellular stress and innate immune responses. Since each RNA platform technology possesses intrinsic strengths and weaknesses, sequence engineering approaches can be combined with platform selection to leverage their capabilities to best suit particular biomedical applications.

## Supplementary Material

Supplement 1

2

## Data Availability

All Supplementary Data are available Online. The underlying replicate data for the experiments performed in this study will be made available on Figshare after peer review.
