# Supplementary material for: Codon optimality modulates cellular stress and innate immune responses triggered by exogenous RNAs
Source: bioRxiv. 2024 Nov 26:2024.11.26.625518. Preprint. [Version 1] doi: 10.1101/2024.11.26.625518 (PMC11623643; doi:10.1101/2024.11.26.625518)
Supplement: Supplement 1 [file media-1.pdf]

Codon optimality modulates cellular stress and innate immune responses triggered by exogenous RNAs

Chotiwat Seephetdee[1,2,3,6] and Daniel L. Kiss[1,2,3,4,5,6,#]

[1]Center for RNA Therapeutics, [2]Department of Cardiovascular Sciences, [3]Houston Methodist Academic Institute, [4]Weil Cornell Medical College, [5]Houston Methodist Cancer Center, [6]Houston Methodist Research Institute, 6670 Bertner Ave, Houston, TX 77030 USA, [#]Corresponding author

To whom correspondence should be addressed:
Daniel L. Kiss, Ph.D.
Center for RNA Therapeutics
Houston Methodist Research Institute
6670 Bertner Ave
R10-113
Houston, TX 77030 USA
Email: dlkiss@houstonmethodist.org

Contact information for all authors:
Chotiwat Seephetdee
Email: chotiwat.seephetdee@gmail.com; orcid.org/0000-0001-7266-4744
Daniel L. Kiss
Email: dlkiss@houstonmethodist.org; orcid.org/0000-0001-5033-7160

Supplementary Tables

Table S1: Key Resources

Reagents or resources	Source	Identifier (Cat. No.)
**Antibodies**		
α-phosphorylated eIF2α (Ser51)	Cell Signaling Technology	3398S
α-eIF2α	Cell Signaling Technology	5324S
α-alpha tubulin	Proteintech	66031-1-Ig
HRP-conjugated goat anti-rabbit IgG	Cell Signaling Technology	7074S
HRP-conjugated horse anti-mouse IgG	Cell Signaling Technology	7076S
**Bacterial strains**		
NEB 5-alpha competent *E. coli*	NEB	C2987U
NEB stable competent *E. coli*	NEB	C3040H
**Reagents**		
NEBuilder HiFi DNA Assembly master mix	NEB	E2621S
Monarch Plasmid Miniprep Kit	NEB	T1010L
HiScribe® T7 mRNA Kit with CleanCap Reagent AG Kit	NEB	E2080S
HiScribe T7 High Yield RNA Synthesis Kit	NEB	E2040S
N^1-methylpseudo-UTP	ThermoFisher Scientific	NU505201
DNase I	NEB	M0303S
Monarch Spin RNA Cleanup Kit	NEB	T2040L
RNase R	Lucigen Corporation	RNR07250
FlashGel RNA Cassettes	Lonza	57027
100X Pen/Strep	Gibco	15140-122
DMEM, high glucose	Gibco	11965092
FBS	Corning	35-075-CV
McCoy's 5A	Gibco	16600-082
Phosphate Buffered Saline (PBS)	GenClone	25-507B
OptiMEM I (1X) Reduced Serum Media	Gibco	11058-021
Trypsin-EDTA 0.25%	Gibco	25200-056

DMSO	Sigma	D4540
Lipofectamine MessengerMAX Transfection Reagent	ThermoFisher Scientific	LMRNA015
Passive Lysis Buffer	Promega	E1941
Dual-Glo Luciferase Assay System	Promega	E2920
Nano-Glo Dual-Luciferase Reporter Assay System	Promega	N1620
Cycloheximide (CHX)	Sigma	C1988
MG-132	Sigma	474787
Pierce BCA protein assay kit	ThermoFisher Scientific	23225
Laemmli sample buffer	Bio-Rad	1610747
12% Mini-PROTEAN TGX Precast Protein Gel	Bio-Rad	4561043
Tris/Glycine/SDS buffer	Bio-Rad	1610732
Trans-blot Turbo Transfer System	Bio-Rad	1704272
TBST	APEX	18-235B
BSA	ThermoFisher Scientific	BP9706-100
Halt Protease Inhibitor Cocktail (100X)	ThermoFisher Scientific	78430
Phosphatase Inhibitor Cocktail II	Sigma	P5726
Phosphatase Inhibitor Cocktail III	Sigma	P0044
PMSF 0.1M	Sigma	93482-50ml-F
NP-40 (10%)	ThermoFisher Scientific	28324
Precision Plus Protein Dual Color Standards	Bio-Rad	1610374
Clarity Western ECL Substrate	Bio-Rad	1705061
TRI Reagent	Zymo Research Corporation	R2050-1-200
Chloroform	Sigma	C2432
Random hexamer primers	Promega	C1181
ProtoScript II Reverse Transcriptase	NEB	M0368S
SSOAdvanced Universal SYBR Green Supermix	Bio-Rad	1725274
**Cell lines**		
BJ	ATCC	CRL-2522
Flp-In T-Rex HEK293	Gibco	R78007

NIH/3T3	ATCC	CRL-1658
U2OS	ATCC	HTB-96
**Software and web server**		
GraphPad Prism 10.1.2		
ImageLab v.4.1		
Incucyte 2022B Rev2		
RNAfold 2.7.0		
https://www.biologicscorp.com/tools/CAICalculator/		

Table S2: Oligonucleotide primers used in this study

Primer name	Sequence	Purpose
CS31	TATGCGTTACCGGCGAGACGCTAC	PCR cloning
CS32	CCTCTTTCAAGCTAAGTGGTATAAACCC	PCR cloning
CS33	GGGTTTATACCACTTAGCTTGAAAGAGG	PCR cloning
CS34	GTAGCGTCTCGCCGGTAACGCATA	PCR cloning
IFI44 Forward	TGGGAGCTGGACCCTGTAAA	qPCR
IFI44 Reverse	CCTCCCTTAGATTCCCTATTTGCT	qPCR
DDX60 Forward	CCGAGGAAGGAAAATGTCGC	qPCR
DDX60 Reverse	CTCACGCAAGGAAACACTGATA	qPCR
IFIT2 Forward	GCACTGCAACCATGAGTGAGA	qPCR
IFIT2 Reverse	GCCTCGTTTTGCCCTTTGAG	qPCR
ISG15 Forward	CCAGGATGCTCAGAGGTTCG	qPCR
ISG15 Reverse	GGGACCTGACGGTGAAGATG	qPCR
RSAD2 Forward	CTCTGTGGAGGAGCCTGGTC	qPCR
RSAD2 Reverse	AAGTTGATCTTCTCCATACCAGCTT	qPCR
TNF-α Forward	TCCCCAGGGACCTCTCTCTA	qPCR
TNF-α Reverse	AGGGTTTGCTACAACATGGGC	qPCR
IL-6 Forward	AGCCACTCACCTCTTCAGAAC	qPCR
IL-6 Reverse	GCCTCTTTGCTGCTTTCACAC	qPCR
RIG-I Forward	TGTGGGCAA TGTCA TCAAAA	qPCR
RIG-I Reverse	GAAGCACTTGCTACCTCTTGC	qPCR
MDA5 Forward	GGCACCATGGGAAGTGATT	qPCR
MDA5 Reverse	ATTTGGTAAGGCCTGAGCTG	qPCR
OAS1 Forward	GCTCCTACCCTGTGTGTGTGT	qPCR
OAS1 Reverse	TGGTGAGAGTACTGAGGAAGA	qPCR
OASL Forward	AGGGTACAGATGGGACATCG	qPCR
OASL Reverse	AAGGGTTCACGATGAGGTTG	qPCR
PKR Forward	TCTTCATGTATGTGACACTGC	qPCR
PKR Reverse	CACACAGTCAAGGTCCTT	qPCR
ACTB Forward	CCCGCGAGCACAGAGCCTCGCCTTTGCCGA	qPCR
ACTB Reverse	CCTTCTGACCCATGCCCACCATCACGCCC	qPCR
