# Supplementary material for: Codon optimality modulates cellular stress and innate immune responses triggered by exogenous RNAs
Source: bioRxiv. 2024 Nov 26:2024.11.26.625518. Preprint. [Version 1] doi: 10.1101/2024.11.26.625518 (PMC11623643; doi:10.1101/2024.11.26.625518)
Supplement: 2 [file NIHPP2024.11.26.625518v1-supplement-2.pdf]

bioRxiv preprint doi: https://doi.org/10.1101/2024.11.26.625518; this version posted November 26, 2024. The copyright holder for this preprint (which was not certified by peer review) is the author/funder, who has granted bioRxiv a license to display the preprint in perpetuity. It is made available under aCC-BY 4.0 International license.

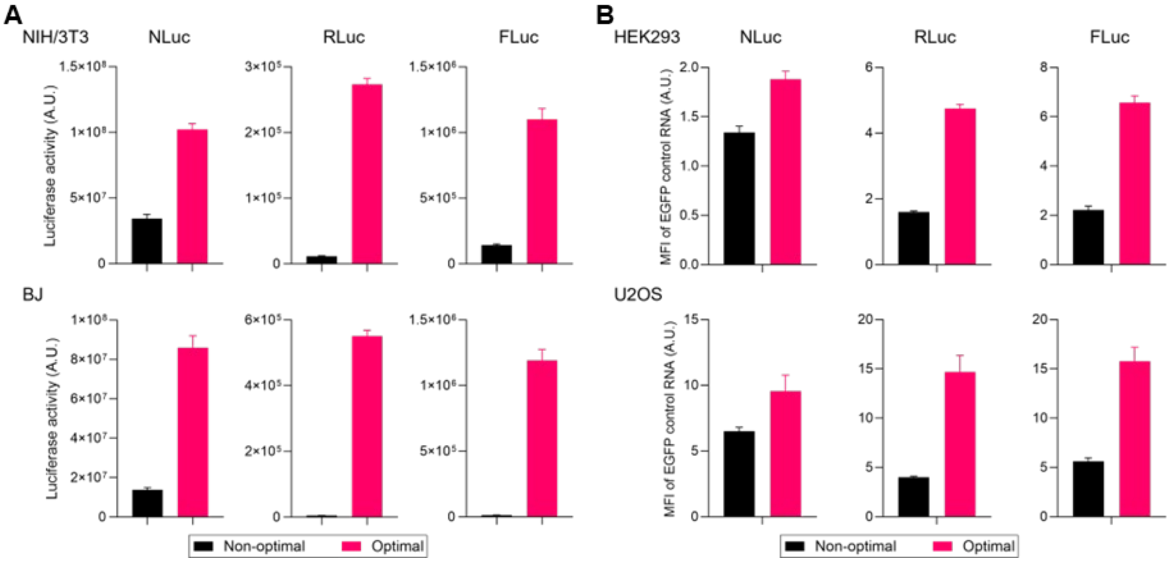

Figure S1. Codon optimality of test mRNAs affects expression of both test and control mRNAs. (A) Dual-luciferase assays using lysates from NIH/3T3 or BJ cells harvested 24 h after transfection with mRNAs encoding either optimal or non-optimal forms of Nanoluciferase (NLuc), *Renilla* luciferase (RLuc), or firefly luciferase (FLuc) reporters (black bars) along with non-optimal luciferase transfection control RNA (pink bars). Luciferase activities of the indicated reporter proteins are shown as the mean +Std Dev of four independent experiments. **(B)** mRNAs encoding luciferase proteins with different codon optimality formulas show differences in protein levels of transfection control mRNAs encoding EGFP. Mean green fluorescence intensity of HEK293 or U2OS cells 24 h after co-transfection with mRNAs encoding either non-optimal (black) or optimal (pink) luciferase reporter and an enhanced green fluorescent protein (EGFP) transfection control. Quantitative fluorescence images were taken by Incucyte S3 and data are shown as the mean signal +Std Dev of four independent replicates with five images taken per replicate.

bioRxiv preprint doi: https://doi.org/10.1101/2024.11.26.625518; this version posted November 26, 2024. The copyright holder for this preprint (which was not certified by peer review) is the author/funder, who has granted bioRxiv a license to display the preprint in perpetuity. It is made available under aCC-BY 4.0 International license.

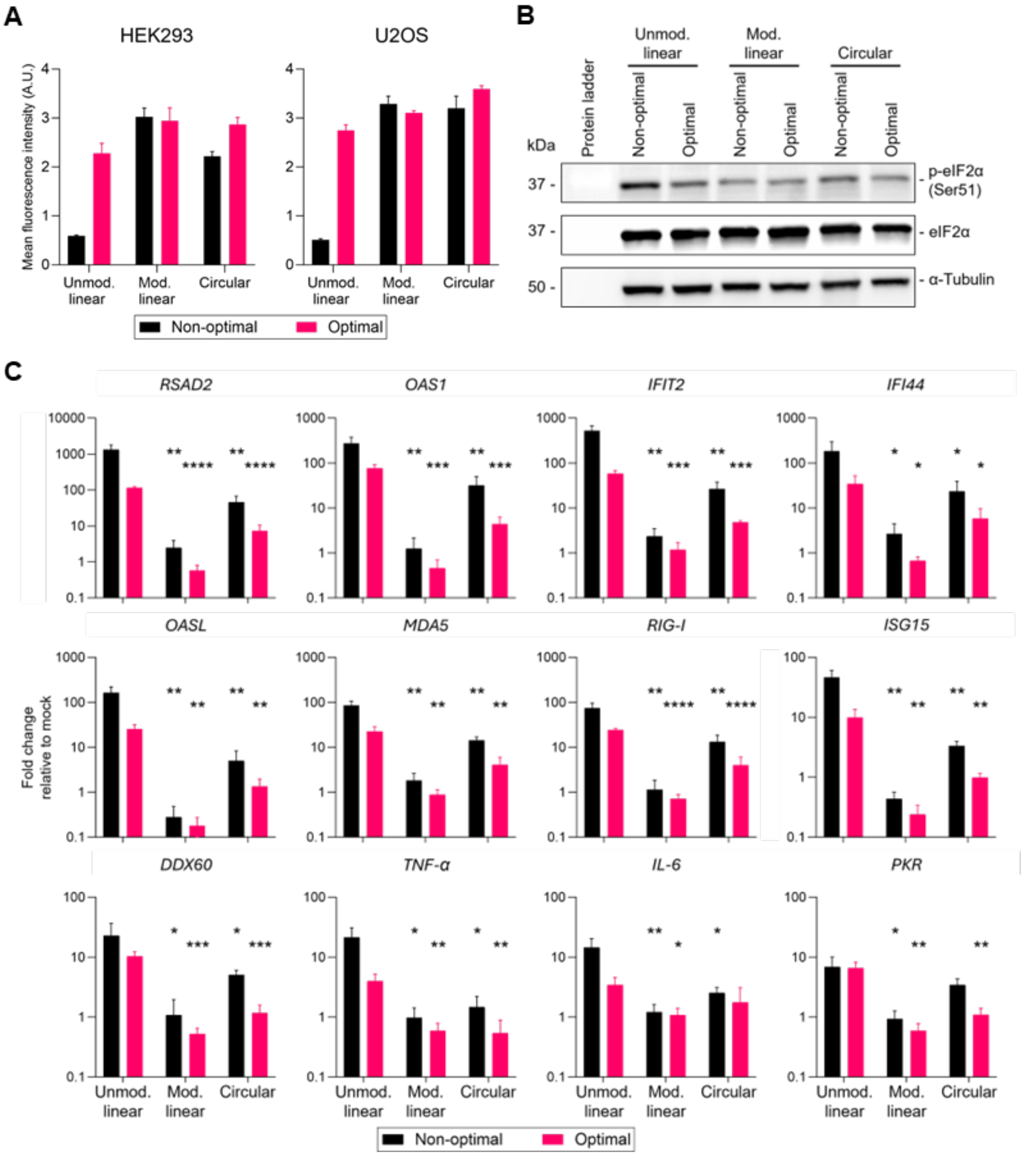

Figure S2. Modified nucleosides and RNA circularization reduce eIF2α phosphorylation and innate immune gene activation. (A) Mean green fluorescence intensity of HEK293 or U2OS cells 24 h after transfection with unmodified linear, modified linear, or circular mRNAs expressing non-optimal (black) or optimal (pink) *Renilla* luciferase reporter along with a green fluorescent protein transfection control. Fluorescence images were taken by an Incucyte S3. Error bars represent Std Dev of four independent replicates with five images taken per replicate. **(B)** Immunoblots were used to assay the phosphorylation of eIF2α in HEK293 cells 16 h after transfection with unmodified linear, modified linear, or circular mRNAs expressing non-optimal or optimal *Renilla* luciferase reporter. Total eIF2α and α-tubulin are used as loading controls. The levels of phosphorylated-eIF2α in each condition were quantified by Image Lab and normalized by eIF2α expression. **(C)** U2OS cells were individually transfected with non-optimal or optimal

bioRxiv preprint doi: https://doi.org/10.1101/2024.11.26.625518; this version posted November 26, 2024. The copyright holder for this preprint (which was not certified by peer review) is the author/funder, who has granted bioRxiv a license to display the preprint in perpetuity. It is made available under aCC-BY 4.0 International license.

Renilla luciferase reporter-encoding unmodified linear, modified linear, or circular RNAs for 16 h. Relative expression of innate immunity genes are measured by qRT-PCR, with relative fold change normalized to expression of control (mock) transfection. Data are shown as the mean of three independent replicates where error bars represent Std Dev. *p < 0.05, **p < 0.01, ***p < 0.001, ****p < 0.0001 compared to unmodified linear with identical coding sequence when using Student's t-test.

bioRxiv preprint doi: https://doi.org/10.1101/2024.11.26.625518; this version posted November 26, 2024. The copyright holder for this preprint (which was not certified by peer review) is the author/funder, who has granted bioRxiv a license to display the preprint in perpetuity. It is made available under aCC-BY 4.0 International license.

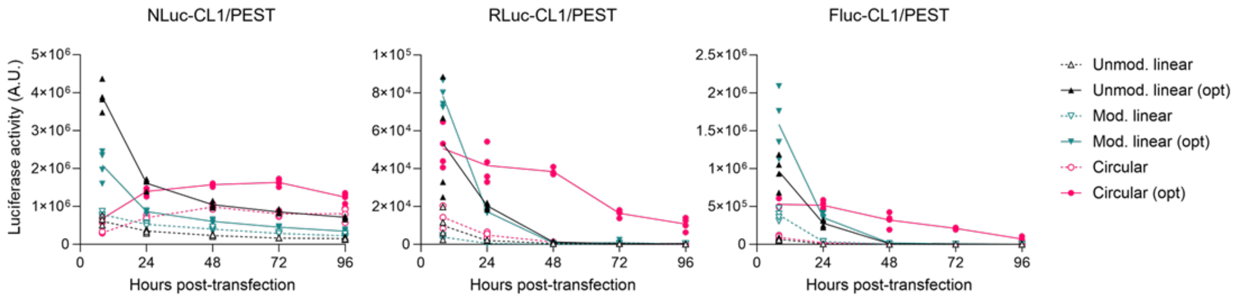

Figure S3. RNA circularization increases the lifespan of optimal reporter transcripts in HEK293 cells. HEK293 cells were co-transfected with a transfection control and either unmodified linear, modified linear, or circular mRNAs encoding non-optimal or optimal CL1/PEST degron-tagged luciferase reporters. Protein expression was measured by the corresponding luciferase assay at 8, 24, 48, 72, and 96 h after transfection. The data are presented as a time-course of luciferase activity from 8 h to 96 h of four independent replicates.

bioRxiv preprint doi: https://doi.org/10.1101/2024.11.26.625518; this version posted November 26, 2024. The copyright holder for this preprint (which was not certified by peer review) is the author/funder, who has granted bioRxiv a license to display the preprint in perpetuity. It is made available under aCC-BY 4.0 International license.

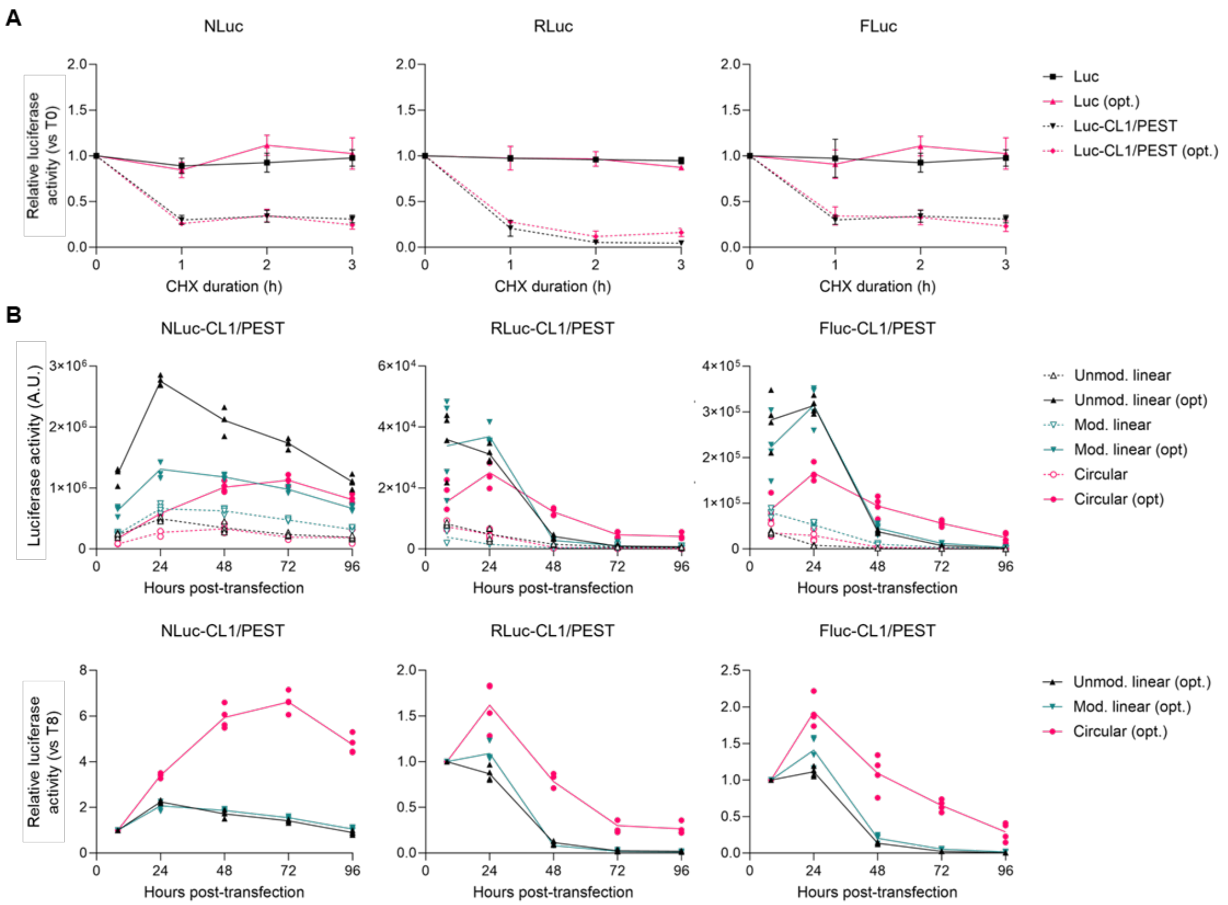

Figure S4. RNA circularization increases the lifespan of optimal reporter transcripts in U2OS cells. (A) U2OS cells were co-transfected with linear mRNAs encoding a transfection control and the indicated reporters for 16 h. Luminescence signals were measured and normalized to the co-transfected control at the indicated time points after addition of cycloheximide (CHX, 100 µg/mL). The data shown for each reporter are the means ±Std Dev of four independent replicates and were compared to the time point when CHX was added (0 h). **(B)** U2OS cells were co-transfected with a transfection control and either unmodified linear, modified linear, or circular RNAs encoding non-optimal or optimal CL1/PEST degron-tagged luciferase reporters. Protein expression was measured by the corresponding luciferase assay at 8, 24, 48, 72, and 96 h after transfection. The data are presented as a time-course of (top) luciferase activity over time from 8 h to 96 h and as a (bottom) relative luciferase activity (RLU) of four independent replicates and were compared to the signal observed 8 h after transfection.

bioRxiv preprint doi: https://doi.org/10.1101/2024.11.26.625518; this version posted November 26, 2024. The copyright holder for this preprint (which was not certified by peer review) is the author/funder, who has granted bioRxiv a license to display the preprint in perpetuity. It is made available under aCC-BY 4.0 International license.

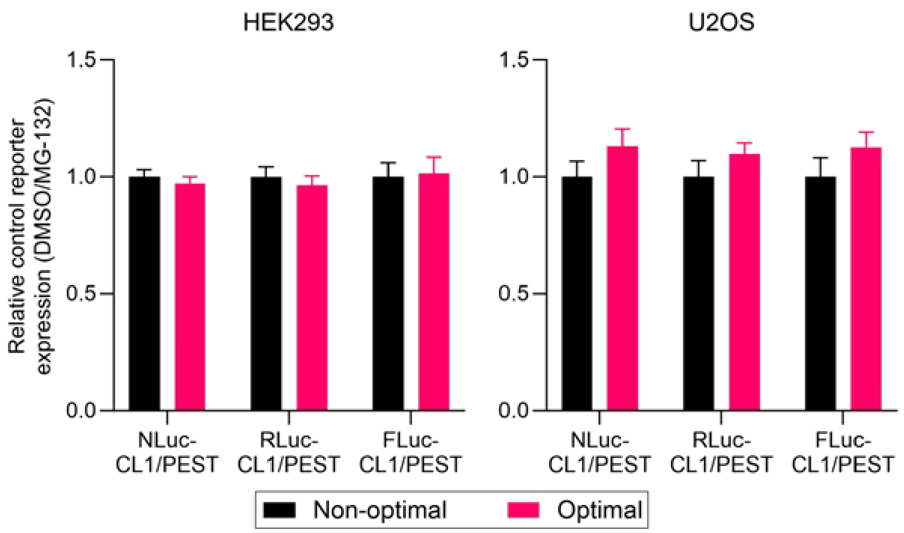

Figure S5. Codon optimality of test mRNAs does not affect proteasome-mediated degradation. HEK293 or U2OS cells were transfected with unmodified linear mRNAs encoding control reporters. After 16 h, cells were subsequently transfected with either non-optimal or optimal CL1/PEST degron-tagged reporters. Cells were then treated with DMSO or 10 μg/mL MG-132 to prevent proteasome-mediated degradation, and luminescence signals were measured 6 h later. The luminescence signals of DMSO-treated cells were normalized to those of MG-132 treated cells. The data shown for each reporter were set relative to the non-optimal reporter and are shown as mean ± Std Dev of four independent replicates.
